# 成人急性淋巴细胞白血病中JAK/STAT信号通路突变患者的预后分析

**DOI:** 10.3760/cma.j.issn.0253-2727.2021.07.011

**Published:** 2021-07

**Authors:** 文娟 樊, 婷婷 徐, 洁洁 郭, 亚飞 李, 中兴 姜

**Affiliations:** 1 郑州大学第一附属医院血液科 450000 Department of Hematology, The First Affiliated Hospital of Zhengzhou University, Zhengzhou 450000, China; 2 河南省人民医院输血科，郑州 450000 Department of Blood Transfusion, Henan Provincial People's Hospital, Zhengzhou 450000, China; 3 郑州大学第一附属医院血液科实验室 450000 Department of Hematology Laboratory, The First Affiliated Hospital of Zhengzhou University, Zhengzhou 450000, China

急性淋巴细胞白血病（ALL）是以原始幼稚淋巴细胞异常增殖为特征的异质性血液系统恶性肿瘤，具有独特的遗传学特征[1]。目前ALL患者的预后影响因素主要集中在血液学、临床特征和遗传因素上，如初诊时WBC、年龄、治疗反应、细胞遗传学和分子学改变以及微小残留病（MRD），后两者与疾病预后密切相关[2]–[3]。近年来，四色流式细胞术、二代测序技术（NGS）的开展极大地提高了分子遗传学改变的检测能力，本研究通过分析246例ALL患者JAK/STAT信号通路突变，以期发现新的有预后价值的基因和新的靶向治疗策略。

## 病例与方法

1. 研究对象：2017年1月至2019年12月就诊于我院血液科的初治ALL患者246例，包括B-ALL 202例，T-ALL 40例，混合表型4例，其中男138例，女108例。所有患者均经分子生物学、免疫学、细胞形态学和遗传学明确诊断，并且符合2016年WHO分型标准。骨髓标本的采集入组患者均知情且签订知情同意书。

2. 主要试剂和仪器：人淋巴细胞分离液为美国Qiagen公司生产；DNA提取试剂盒为中国天根生化科技公司生产；Miseq二代测序仪为美国Illumina公司产品；FACSCanto流式细胞仪为美国Becton Dickinson公司产品。

3. 白血病免疫分型及MRD检测：采用叶绿素蛋白（pr5eCP）、异硫氰酸荧光素（FITC）、别藻蓝素（APC）、藻红蛋白（PE）四种免疫荧光染料标记美国BD公司生产的单克隆抗体，B-ALL、T-ALL及MRD检测的抗体选择参照文献[4]，具体操作步骤同文献[5]。使用FACSCanto流式细胞仪分析20 000个细胞，以CD45/SSC双参数设门，原始幼稚细胞≥20％为阳性。

4. NGS检测：无菌操作环境中，用EDTA-K_2_凝抗管收集骨髓液标本2 ml。采用人淋巴细胞分离液收集单个核细胞，随后进行细胞总DNA提取及文库构建。应用NGS共检测16种ALL相关突变基因，包括NT5C2、CREBBP、PHF6、CRLF2、PAX5、JAK1、NOTCH1、PTEN、IL-7R、FBXW7、SH2B3、TP53、JAK3、FLT-3、JAK2、IKZF1。

5. 治疗方案、疗效评价及随访：246例初治ALL患者，其中7例拒绝治疗出院，其他患者根据情况以VDC（L）P（长春地辛、柔红霉素、环磷酰胺、门冬酰胺酶、地塞米松）为基础方案化疗，后续给予Hyper-CAVD（环磷酰胺、吡柔比星、长春地辛、地塞米松）、CAM（环磷酰胺、阿糖胞苷、6-巯基嘌呤）等方案。BCR-ABL阳性者加用口服达沙替尼等酪氨酸激酶抑制制。参照《血液病诊断及疗效标准》（第4版）评价疗效。MRD>0.0001为阳性。

6. 随访：随访截止日期为2020年1月31日，采用查阅病例及电话的方式随访，中位随访时间7（1～35）个月。失访30例（包含未治疗的7例），失访率为12.2％。无疾病进展生存（PFS）期为确诊日至疾病进展或死亡的时间，总生存（OS）期为确诊日至死亡或者末次随访的时间。

7. 统计学处理：采用SPSS 21.0统计软件分析数据，计数资料的比较采用*χ*^2^检验，生存分析采用Kaplan-Meier法，*P*<0.05为差异有统计学意义。

## 结果

1. 一般特征：246例成人ALL患者中，初诊首发症状以发热为主（66％），其次为乏力（35％）、出血（20％）。肝脾肿大者111例（44.9％），淋巴结肿大者100例（40％）。

2. 基因突变情况：通过NGS共检出69例患者伴基因突变,总突变率为28.0％，共12种突变基因，T-ALL（70.0％）较B-ALL（18.8％）患者更易发生基因突变（*P*<0.05）。突变基因详见表1。在T-ALL中突变较频繁的基因依次为NOTCH1、JAK3、FBXW7、IL-7R；B-ALL中突变较频繁基因为TP53、IL-7R、PAX5。

**表1:** 成人急性淋巴细胞白血病（ALL）突变频率较高的基因及其突变率［例（％）］

突变基因	B-ALL（202例）	T-ALL（40例）	T/B-ALL（3例）
NOTCH1	2（1.0）	16（36.4）	0（0）
IL-7R	9（4.5）	5（12.5）	0（0）
TP53	11（5.4）	1（2.5）	0（0）
JAK3	1（0.5）	8（20.0）	1（33.3）
FBXW7	0（0）	5（12.5）	1（33.3）
FLT-3	4（2.0）	2（5.0）	0（0）
PAX5	5（2.5）	0（0）	0（0）

所有发生基因突变的ALL中27.5％（19/69）的患者发生基因共突变（即同时发生2个或2个以上的基因突变），其中84.2％（16/19）涉及JAK/STAT信号通路（表2）。有2例T-ALL患者发生三种基因共突变：JAK3、TP53和PHF6共突变1例，JAK1、NOTCH1和FBXW7共突变1例；另外，1例双表型患者发生JAK3、JAK1和FBXW7共突变。余为NOTCH1和FBXW7共突变、NOTCH1和TP53共突变、PAX5和CREBBP共突变各1例。共突变之间存在基因互斥和共存现象，NOTCH1、FBXW7基因与其他基因存在共存，而IL-7R与JAK3和JAK1之间存在互斥。

**表2:** 成人急性淋巴细胞白血病（ALL）中JAK/STAT信号通路基因共突变情况（例）

突变基因	NOTCH1	FBXW7	PHF6	SH2B3
JAK3				
T-ALL	3	1	3	0
B-ALL	1	0	0	0
IL-7R				
T-ALL	3	0	0	0
B-ALL	0	0	0	1
JAK1				
T-ALL	2	1	0	0
B-ALL	0	0	0	0

另外，本研究发现所有发生共突变的基因中与JAK3基因发生共突变的最多，10例JAK3突变患者中有9例发生了基因共突变，其临床特征见表3。

**表3:** JAK3基因突变急性淋巴细胞白血病（ALL）患者的临床特征和基因突变情况

例号	外显子	突变位点	突变频率（％）	年龄（岁）	性别	类型	WBC（×10^9^/L）	共突变基因	融合基因	转归
1	13	A573V	28.81	23	男	T-ALL	1.2	TP53、PHF6	阴性	死亡
	15	R657Q	19.23							
2	11	M511I	46.68	21	女	T-ALL	4.9	NOTCH1	阴性	死亡
3	15	R657Q	32.00	17	男	B-ALL	96.6	NOTCH1	阴性	死亡
4	11	M511I	6.81	38	男	T-ALL	317.1	NOTCH1	阴性	死亡
5	15	R657Q	85.28	23	男	T-ALL	6.9	NOTCH1	SET-CAN	完全缓解
6	11	M511I	90.08	16	男	T/B-ALL	150.5	JAK1、FBXW7	阴性	完全缓解
7	15	R657Q	14.45	14	女	T-ALL	5.7	FBXW7	阴性	完全缓解
8	15	V674A	7.08	18	男	T-ALL	93.8	PHF6	SET-CAN	完全缓解
9	15	V674A	69.18	21	男	T-ALL	11.2	PHF6	TLS-ERG	完全缓解
10	15	R657Q	15.61	17	男	T-ALL	158.0	无	阴性	完全缓解

3. 疗效分析：本研究中涉及较多的信号通路包括JAK/STAT信号通路（JAK1、JAK3、IL-7R）和NOTCH信号通路（NOTCH1、FBXW7）。JAK/STAT信号通路突变的患者在诱导治疗时完全缓解（CR）率低于阴性组，差异无统计学意义，但诱导治疗后MRD的阳性率却较阴性组高，差异有统计学意义（*P*<0.05）。在T-ALL中NOTCH信号通路突变的患者诱导治疗的CR率与阴性组相似，但MRD阳性率低于阴性组，差异无统计学意义（表4）。因NOTCH信号通路突变在B-ALL中发生率极低，未进行分析。

**表4:** 信号通路突变对急性淋巴细胞白血病（ALL）患者初始治疗疗效的影响［例（％）］

疗效	所有ALL患者			T-ALL患者		
	JAK/STAT通路突变阳性（25例）	JAK/STAT通路突变阴性（214例）	*P*值	NOTCH1突变阳性（18例）	NOTCH1突变阴性（21例）	*P*值
CR	16（64.0）	153（71.5）	0.225	13（72.2）	15（71.4）	0.876
NR/PR	9（36.0）	61（28.5）		5（27.8）	6（28.6）	
MRD阳性	17（68.0）	97（45.3）	<0.001	8（44.4）	12（57.1）	0.066
MRD阴性	8（32.0）	117（54.7）		10（55.6）	9（42.9）	

注：CR：完全缓解；PR：部分缓解；NR：未缓解；MRD：微小残留病

本研究进一步对216例可随访的患者进行生存分析，JAK/STAT通路突变阳性组的中位OS期（12个月对13个月）及中位PFS期（6个月对9个月）均低于阴性组，但差异无统计意义，由于随访时间尚短，需进一步随访。

## 讨论

研究表明NOTCH信号通路能激活JAK-STAT信号通路和PI3K/AKT信号通路[6]。较高的突变频率及其在调节ALL细胞存活和增殖中的中心作用，使NOTCH1成为T-ALL治疗的重要靶标[7]。具有NOTCH1和（或）FBXW7突变的患者比没有这些突变的患者早期治疗反应好，且具有更好的OS和PFS[8]，但是国内的一项研究显示，NOTCH1突变与预后无关[9]。目前MRD是ALL患者较强的预测因子[10]–[11]。

JAK/STAT信号通路突变在ALL中很常见，在B-ALL中发生率为11％[12]，在T-ALL中更高，本研究中JAK/STAT通路突变在T-ALL中的发生率为40％。IL-7R的激活导致JAK/STAT信号通路的磷酸化，从而介导了白血病细胞对糖皮质激素的抵抗，早期治疗反应差。本研究中早期诱导治疗的CR率低于阴性组，而MRD阳性率更是明显高于阴性组，提示预后可能差。有研究表明，在BCR-ABL阴性的B-ALL患者中JAK/STAT信号通路突变的患者预后差[13]，其研究主要涉及JAK1、JAK2突变基因。本研究中，JAK/STAT信号通路突变涉及的基因是IL-7R和JAK3。在难治/复发的T-ALL的RNA测序中发现，JAK/STAT和RAS/PTEN信号通路是耐药或早期复发最常见的途径，通常与NOTCH1/FBXW7突变相关，而JAK/STAT通路中以JAK3的突变率最高。在仅携带NOTCH1/FBXW7突变的患者中观察到明显更好的生存，但伴随这些通路突变时则消除了这种有利的预后效果[14]。本研究中JAK3突变与NOTCH1共突变的患者最多，其中3例患者目前死亡，预后极差，另1例存活的患者2个疗程化疗后MRD均为阳性。与FBXW7共突变的患者有2例，目前均为CR。截至末次随访日期生存时间最长为11个月。一项81例成人T-ALL患者的二代测序研究中也发现JAK3突变多发生于高危T-ALL患者[15]。

本研究中，在ALL中共发现4种JAK3的突变位点，其中3种突变位点位于JH2区：A573V、R657Q、V674A。T细胞幼淋巴细胞白血病（T-cell prolymphocytic leukemia，T-PLL）是一种罕见的以成熟的T细胞为特征的老年性疾病，治疗效果差且常面临复发，预后不良。T-PLL中JAK1/JAK3突变频率很高，其中M511I是最常检出的位点，其突变位于SH2结构域和JH2结构域之间[16]–[18]。Bergmann等[16]发现同时携带V674F和V678L的突变体及M511I和R657Q突变体。Bellanger等[17]发现同时携带M511I和A572V的突变体。本研究中我们发现1例A573V和R657Q同时突变的患者，该患者化疗获得CR，但MRD阳性，最终因感染性休克死亡。研究表明M511I突变在T-ALL中最常见，在34.7％的JAK3突变患者中，检测到M511I突变以及JAK3的第二个突变。两个JAK3突变或JAK3和JAK1共突变，一个JAK3突变通常是主要克隆，第二个突变则是次要克隆[19]。与单独的突变相比，多个JAK3突变的激活能力更强[20]。JAK3 M511I和JAK3 A573V在早期前体T淋巴细胞白血病（ETP-ALL）中是突变率较高的位点[21]。而本研究中检测到最多的突变位点为R657Q，约占50％，其次为M511I（30％）。全基因组分析显示JAK3 V674A突变可能与疾病的复发密切相关[22]。本研究中有2例患者出现V674F突变，1例诱导治疗时获得部分缓解（PR），另1例获得CR，但MRD阳性，目前2例患者均为CR状态，需继续随访。JAK3突变在B-ALL中罕见，Mullighan等[23]在儿童高危ALL中发现1例JAK3 S789P突变的B-ALL患者。我们在研究中发现1例B-ALL患者，突变位点为R657Q，该患者入院1周即死亡，预后极差。在儿童ALL中JAK3突变率最高的位点为JAK3 V722I，且可能与疾病复发有关。

既往研究表明，IL7R-JAK信号传导途径（IL-7R、JAK1、JAK3）与表观遗传修饰因子（WT1、PHF6、PRC2）之间存在功能性相互作用[19]。据报道PHF6突变常伴随SET-CAN、TLS-ERG融合基因表达，PHF6是X连锁的，几乎只在男性中出现突变，与预后不良相关[24]。本研究中3例JAK3突变的患者合并PHF6突变，均为男性，其中2例分别表达SET-CAN、TLS-ERG融合基因，目前均持续CR，但诱导治疗后MRD均为阳性，另1例患者死于严重感染，诸多因素可能提示患者预后不佳，但随访时间尚短。本研究中JAK3突变患者共计10例，诱导治疗中反应相对较好，1例死亡，1例PR，其余均为CR，但是MRD阳性率却远高于JAK3突变阴性组，提示JAK3突变的患者预后差。

综上所述，在T-ALL中普遍存在基因突变和共突变，而对一些基因的预后意义尚未达成共识。
